# Retrobulbar Optic Neuritis Post Typhoid fever: Atypical Case Report

**DOI:** 10.22336/rjo.2023.13

**Published:** 2023

**Authors:** Aparajita Chaudhary, Kriti Bhatt, Shalini Verma, Apurva Bagla, Vijay Kumar Maurya

**Affiliations:** * Department of Ophthalmology (Regional Institute of Ophthalmology), M.L.N. Medical College, Prayagraj, Uttar Pradesh, India

**Keywords:** Retrobulbar Optic Neuritis, typhoid fever

## Abstract

**Purpose:** Post typhoid autoimmune-mediated simultaneous retrobulbar optic neuritis (RBN) involving both eyes is a rare complication requiring early diagnosis and prompt treatment.

**Case presentation:** We present a case of bilateral RBN in a six-year-old male who came to our department with a chief complaint of sudden onset painless profound loss of vision in both eyes, after an episode of high-grade fever 2 weeks earlier. Perception of light was doubtful in right eye (RE) and vision was hand movement in left eye (LE). On ocular examination, anterior segment and fundoscopy of both eye were normal. Blood investigation was normal except for raised ESR. CT of brain and orbit was normal. MRI of brain and orbit revealed bilateral thickening and restriction of optic nerve suggestive of ON. He was initiated with intravenous methyl-prednisolone for three consecutive days after which tapering doses of oral corticosteroid was given.

**Results:** A rapid and marked improvement in Uncorrected Visual Acuity (UCVA) was observed with UCVA improving to 6/ 12 RE and 6/ 9 LE post 1 month. The pupillary reaction also became normal in both eyes. Moreover, there was a significant reduction in the Widal titre of the patient post 2 weeks of treatment.

**Discussion:** Paediatric ON has rare and unique characteristics, which differentiates it from adult ON. No clinical trials have been performed for paediatric ON, so current clinical practice follows the evidence drawn from the Optic Neuritis Treatment Trial (ONTT).

**Conclusion:** Paediatric ON is uncommon. Despite having clinically severe bilateral vision loss, retrobulbar optic neuritis in children post typhoid fever has excellent response to steroid therapy if early diagnosed and treated.

**Abbreviations:** RBN = Retrobulbar Optic Neuritis, MRI = Magnetic Resonance Imaging, CT = Computerized Tomography, UCVA = Uncorrected Visual Acuity, RE = Right eye, LE = Left eye, ON = Optic neuritis, ONTT = Optic Neuritis Treatment Trial

## Introduction

ON refers to the swelling of the optic nerve. This disease is rare in children and behaves very differently in children compared to adults. Children are more prone to develop bilateral vision loss, anterior involvement of 2nd cranial nerve with papillitis, an associated viral, bacterial, or Para infectious aetiology; and better visual prognosis compared to adults [**1**-**4**].

Para infectious optic neuritis is ON arising as a result of infectious aetiology developing days-week post infection. It occurs either due to direct invasion by pathogen or post infectious disease, presumably autoimmune-mediated demyelination or inflammation of the optic nerve [**5**]. Salmonella typhi, a common gastrointestinal pathogen, causes typhoid fever that affects the gastrointestinal tract, joints and heart. It rarely affects the eye. Retinitis, vasculitis, optic nerve and subsequent macular involvement are the various forms of posterior segment involvement [**6**]. 

We report an interesting case of bilaterally involved Retrobulbar Optic Neuritis in a 6-year male following high grade fever secondary to Salmonella typhi infection, 15 days prior to presentation, managed successfully with corticosteroid therapy.

## Case Report

A 6-year-old male came to our department with simultaneous sudden onset painless profound vision loss in both eyes for 3 days. His parents offered information on his history of high-grade fever and vomiting 15 days before and his admission elsewhere for the same medical problem, where he was diagnosed with Salmonella typhi fever. He was conservatively managed with antipyretic and antibiotic medication. The patient had no history of recent injury to the head, recurrent redness of the eye, diplopia and vaccination. No significant medication history for any systemic disease was registered. Birth history, and growth and development of the child were normal.

General examination: The child was average built, afebrile, fully alert, with normal vitals. Systemic and neurological examination were normal. 

**Fig. 1:**
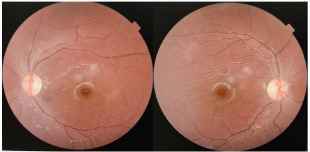
Fundus photograph showing normal optic disk

**Fig. 2:**
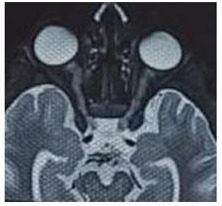
MRI brain and orbit showing bilateral optic nerve thickening suggestive of optic neuritis

Ocular examination: Perception of light was doubtful in the right eye and vision was hand movement in the left eye with ill-sustained pupillary reaction. Extraocular movements were normal. Anterior and posterior segment and dilated fundoscopic examination were unremarkable in both eyes (**Fig. 1**). Blood test result showed total leucocyte count 19,100 (N87 L11 M1 E1 B0) with ESR 45 mm. Blood sugar, VDRL, Mantoux Test, Liver function test, urine routine microscopy, chest X-ray, were not significant. MRI-brain and orbit revealed bilateral thickening and restriction of optic nerve, suggestive of optic neuritis (**Fig. 2**). 

Treatment: He was started on intravenous methyl-prednisolone 25 mg/ kg/ day for 3 consecutive days followed by oral corticosteroid at a dose of 1 mg/ kg/ day tapered gradually over a period of 4 weeks (after paediatrician consultation). 

## Results

Post initiation of treatment on the 4th day, his UCVA was 3/ 60 - right eye (RE) and 6/ 60 - left eye (LE), which improved to 6/ 18 RE and 6/ 12P LE after 2 weeks. The pupillary reaction also became normal in both eyes. The pupillary reaction also became normal in both eyes. Moreover, a significant reduction in the Widal titre of the patient was observed after 2 weeks. The patient was regularly followed and his UCVA was 6/ 12 RE and 6/ 9 LE after 1 month.

## Discussion

Optic neuritis refers to inflammation or demyelination of the optic nerve, characterized by visual impairment, associated with ocular pain and dyschromatopsia. It is associated with an infectious cause, para infectious process, CNS demyelinating condition, post-immunization, or can present as an idiopathic condition. Other causes include adjacent inflammation of paranasal sinus, systemic collagen vascular diseases and intraocular inflammation [**7**,**8**]. 

Paediatric ON has rare and unique characteristics of paediatric optic neuritis that differentiate it from adult ON, which include: (1) anterior involvement, with optic disc swelling seen in ≥ 70% of cases [**3**], (2) presents as simultaneous bilateral condition (in up to 60% cases); (3) often occurs within 1-2 weeks after a confirmed/ presumed infection or post-vaccination; (4) is often less associated with Multiple Sclerosis (15%-44% cases); and (5) is corticosteroid sensitive and corticosteroid dependent. What is also important about it is the absence of relative afferent pupillary defect in symmetric bilateral cases. 

Typically, para infectious ON occurs following the onset of infectious aetiology (viral, or less common bacterial), by 1-3 weeks. Its pathogenesis is due to an autoimmunologic-inflammatory mechanism. Rapopport et al. reported that time period elapsed between the onset of the visual symptoms and the febrile illness was much shorter in the paediatric age group. This can be due to a more fulminant immune response in children, manifested in the form of bilaterally involved optic nerve and other neurological symptoms, with or without magnetic resonance imaging (MRI) abnormalities [**9**,**10**]. 

No clinical trials have been performed for paediatric ON, so current clinical practice follows the evidence drawn from the Optic Neuritis Treatment Trial (ONTT). Paediatric ON patients are treated based on the analogous treatment regimen as adults, integrating a weight-adjusted dosing of intravenous corticosteroids, followed by oral corticosteroids. A typical dose ranges from 4 to 30 mg/ kg/ day, with a maximum dose of 1 g/ day, for 3 to 5 days. Most of the children are treated with 1 mg/ kg/ day oral dose tapered slowly over 4 to 6 weeks to avoid relapse if tapered quickly [**9**,**11**-**15**]. 

We treated the child with intravenous methyl-prednisolone 30 mg/ kg/ day for 3-consecutive days followed by oral corticosteroid at a dose of 1 mg/ kg/ day tapered slowly over a period of 4 weeks (after paediatrician consultation). On the 4th day, his UCVA was 3/ 60 RE and 6/ 60 LE, which improved to 6/ 18 RE and 6/ 12P LE after a period of 2 weeks. Also, there was a significant reduction in the Widal titre of the patient after 2 weeks. The patient was regularly followed and his UCVA was 6/ 12 RE and 6/ 9 LE after 1 month. 

Although visual acuity at the moment of presentation tends to be poor in children compared to adults, they show more remarkable responses to corticosteroid therapy than adults. Brady et al. [**1**] reviewed 25 children having ON and stated that children are more prone to develop bilateral disease and have a good visual prognosis, although approximately 20% of the eyes remain visually impaired. A normal MRI is associated with a better prognosis [**16**]. Wilejto et al. examined 36 children suffering from ON in Canada and reported that in 83% of cases, visual acuity recovered to ≥ 20/ 40 after a follow-up of 2.4 years [**17**]. 

It is of utmost importance to follow the patients with ON during series of examinations and MRIs after an initial episode of ON because the risk of recurrence of ON neurological disability is dependent on the diagnosis of the child who has an underlying neurological disorder (such as multiple sclerosis or neuromyelitis optica).

## Conclusion

Optic neuritis is not common in the paediatric age group. Para infectious optic neuritis usually presents 1 to 3 weeks following the onset of infectious aetiology (viral or bacterial infection). It is usually diagnosed based on clinical features of the ON, history of prior infection, and lab test underlying particular infections. According to ONTT, optic neuritis in children is treated in the similar manner, with steroids, as in adults. Paediatric ON has better visual prognosis compared to adults’ ON, if diagnosed and treated early.

**Conflict of Interest**

The authors state no conflict of interest.

**Informed Consent and Human and Animal Rights statement**

Informed consent has been obtained from the legal guardians of the individual included in the study. 

**Authorization for the use of Human subject**

Ethical approval: The research related to human use complies with all the relevant national regulations, institutional policies, is in accordance with the tenets of the Helsinki Declaration, and has been approved by the review board of M.L.N. Medical College, Prayagraj, Uttar Pradesh, India.

**Acknowledgements**

None.

**Sources of Funding**

None.

**Disclosures**

None. 

**Presentation at a meeting**

NA.
